# Vitamin D Concentrations at Birth and the Risk of Rheumatoid Arthritis in Early Adulthood: A Danish Population-Based Case-Cohort Study

**DOI:** 10.3390/nu14030447

**Published:** 2022-01-20

**Authors:** Isabel Cardoso, Ina Olmer Specht, Fanney Thorsteinsdottir, Marta Jadwiga Thorbek, Amélie Keller, Maria Stougaard, Arieh S. Cohen, Mina Nicole Händel, Lars Erik Kristensen, Berit Lilienthal Heitmann

**Affiliations:** 1 The Parker Institute, Bispebjerg and Frederiksberg Hospital, 2000 Frederiksberg, Denmark; isa.msc@gmail.com (I.C.); fanney.thorsteinsdottir@regionh.dk (F.T.); amelie.keller@sund.ku.dk (A.K.); maria.stougaard@psy.ku.dk (M.S.); mina.nicole.holmgaard.handel@regionh.dk (M.N.H.); lars.erik.kristensen@regionh.dk (L.E.K.); Berit.Lilienthal.Heitmann@regionh.dk (B.L.H.); 2 Center for Neonatal Screening, Department of Congenital Disorders—Clinical Mass Spectrometry, Statens Serum Institute, 2300 Copenhagen, Denmark; MAJB@ssi.dk; 3 Section of Epidemiology, Department of Public Health, University of Copenhagen, 1014 Copenhagen, Denmark; 4 Center for Early Intervention and Family Studies, Department of Psychology, University of Copenhagen, 1353 Copenhagen, Denmark; 5 Clinical Mass Spectrometry, Staten Serum Institute, 2300 Copenhagen, Denmark; ACO@ssi.dk; 6 The Boden Group, Faculty of Medicine and Health, Sydney University, Sydney, NSW 2006, Australia; 7 Section for Clinical Practice, Department of Public Health, University of Copenhagen, 1014 Copenhagen, Denmark

**Keywords:** rheumatoid arthritis, vitamin D, dried blood spots, Denmark, early adulthood, cohort

## Abstract

Background: Low vitamin D in pregnancy may impair the development of the fetal immune system and influence the risk of later development of rheumatoid arthritis (RA) in the offspring. The aim was to examine whether lower 25-hydroxyvitamin D3 (25(OH)D) concentrations at birth were associated with the risk of developing RA in early adulthood. Methods: This case-cohort study obtained data from Danish registers and biobanks. Cases included all individuals born during 1981–1996 and recorded in the Danish National Patient Register with a diagnosis of RA with age >18 years at first admission. The random comparison consisted of a subset of Danish children. Vitamin D concentrations were measured in newborn dried blood. In total, 805 RA cases and 2416 individuals from the subcohort were included in the final analysis. Weighted Cox regression was used to calculate hazard ratio (HR). Results: The median (interquartile rage (IQR)) 25(OH)D concentrations among cases were 24.9 nmol/L (IQR:15.4;36.9) and 23.9 nmol/L (IQR:13.6;36.4) among the subcohort. There was no indication of a lower risk of RA among individuals in the highest vitamin D quintile compared with the lowest (HRadj.:1.21 (0.90;1.63)). Conclusion: The risk of RA in early adulthood was not associated with vitamin D concentrations at birth.

## 1. Introduction

Rheumatoid arthritis (RA) is a chronic autoimmune inflammatory disease that affects primarily the joints [1] and is characterized by chronic inflammation and progressive joint destruction, which often results in physical disability [1,2]. RA is also a systemic disease, because it can affect several other organs, such as, e.g., the heart and lungs; thus, contributing to increased comorbidity and mortally among patients with RA [1,2]. This disease is one of the most common forms of chronic inflammatory arthritis [1], with a prevalence of 0.5–1.0% in developed countries and affecting women more often than men with a peak incidence at age 40–65 years [3]. RA is a complex and multifactorial disease, with both genetic and environmental factors interacting and contributing to the risk of developing the disease [4,5]. Among the environmental factors, vitamin D has been pointed out as a potential risk factor for the development of autoimmune diseases, including RA [6]. Vitamin D is a steroid hormone with immunomodulatory properties that exists in two main forms; vitamin D2 (ergocalciferol), which is obtained from plants, and vitamin D3 (cholecalciferol) which is synthesized de novo in the skin on exposure to ultraviolet-B radiation, and also available from few animal source foods [7].

Maternal vitamin D deficiency is a public health problem, widespread in various populations and latitudes [8,9,10]. Since the fetus’ and newborn’s vitamin D concentrations depend entirely on the mother’s [8,11], maternal vitamin D deficiency is a problem extended to the offspring. Based on the evidence suggesting that low vitamin D in early life impairs the development of the immune system [12,13,14], and that maternal vitamin D deficiency has been shown to influence the spectrum of immune cells in maternal and cord blood [15], it is possible that vitamin D deficiency during pregnancy has programming effects in terms of immune tolerance in the fetus, which can increase the risk of developing autoimmune diseases, such as RA [16].

To date, no studies have been identified that examine whether perinatal vitamin D concentrations influence the risk of developing RA in the offspring later in life. In Denmark, there are good resources to examine such associations, provided by the existence and availability of unique national administrative and health registers and biobank material, with almost complete nationwide coverage.

Therefore, the aim of this study was to examine whether 25-hydroxyvitamin D concentrations (25(OH)D) at birth were associated with the risk of developing RA in early adulthood, age 18 to 33 years. We hypothesized that low concentrations of vitamin D at birth are associated with increased risk of RA in early adulthood.

## 2. Materials and Methods

### 2.1. Study Population and Data Sources

This study is part of the D-tect project, which is registered at www.clinicaltrials.gov (NCT03330301).

This registry-based case-cohort study adhered to the “Strengthening the Reporting of Observational studies in Epidemiology” (STROBE) guidelines (Table S1) [17]. We obtained all relevant data from the Danish health care registers. In Denmark, every citizen receives a unique personal civil registration number (CPR) at the time of birth or immigration. The CPR number is used in all national registries, which makes it possible to accurately link individual-level information [18] and thus follow individuals over time [19]. The Danish National Patient Register (NPR), which contains information about hospital contacts, including date, diagnoses (coded according to the International Classification of Diseases (ICD) tenth revision (ICD-10, after 1994)) and associated procedures [20], was used to identify individuals with RA and retrieve related information (such as recorded date of diagnose). RA cases were defined as all individuals who were born in Denmark during 1 May 1981–31 December 1996, that were recorded in the NPR with an ICD-10 code for RA (M05.0, M05.1D, M05.2, M05.3, M05.8, M05.9, M06, M06.0, M06.1, M06.2, M06.3, M06.4, M06.8, M06.9) after the age of 18 years at first admission (event occurrence period during 1 May 1999–10 April 2015, *n* = 1126). The Danish Civil Registration System (CRS), which is an administrative register that contains information on all persons residing in Denmark, including updated information on vital status and emigration [18,19], was used to identify all liveborn children during 1 May 1981–31 December 1996 (N = 921,804). Among those, a random subcohort was further sampled conditional on the year of birth with more weight to those born during 1981–1985, as more cases were born in these years.

**Figure 1:**
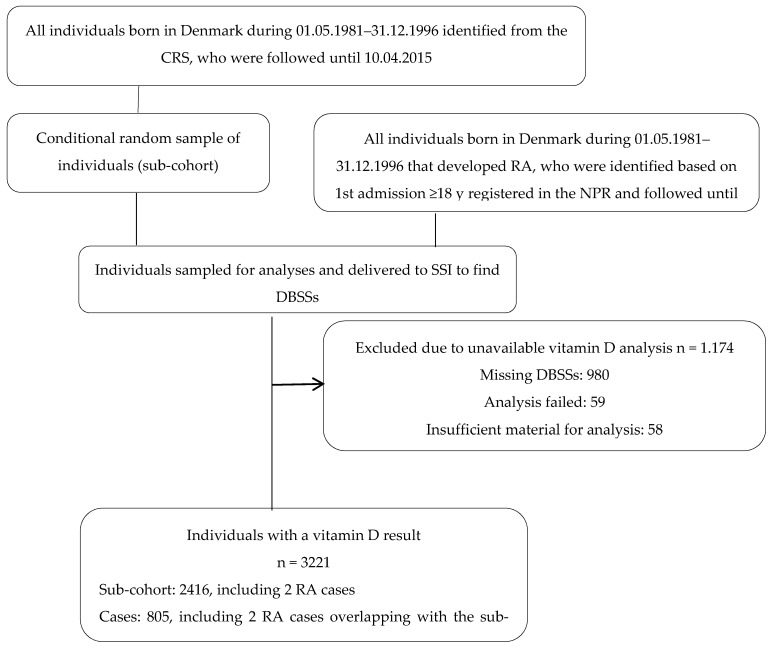
Flowchart of the study population. CRS: Danish Civil Registration System; RA: rheumatoid arthritis; NPR: Danish National Patient Register; SSI: Statens Serum Institute; DBSSs: dried blood spot samples.

Among the individuals in the subcohort, two individuals had developed RA (Figure 1). The analyses were based on complete cases, thus individuals were excluded from the subcohort group and case group if they had missing vitamin D measurement (306 and 791, respectively) or missing information on other covariates (*n* = 77). Thus, 805 cases and 2416 individuals from the subcohort were included in the final analysis (Figure 1).

### 2.2. Assessment of 25(OH)D Concentrations and Data Sources

In Denmark, within the first week of life, all newborns have capillary blood sample collected by heel prick, which is then dehydrated in special filter paper (dried blood spot samples (DBSSs)). DBSSs are used for routine screening of congenital disorders, and after screening, the residual samples are stored at −20 °C in a freezer facility at the Danish Newborn Screening Biobank at Statens Serum Institute (SSI), Copenhagen, Denmark [21]. Evidence indicates that 25(OH)D concentrations from DBSSs and cord blood are highly correlated [22]. Although there are no quality-assurance programs for 25(OH)D measures in DBSSs, the SSI laboratory participates in the Vitamin D External Quality Assessment Scheme with the equivalent-serum method [23]. The determination of neonatal 25(OH)D concentrations was done by measuring 25(OH)D2 and 25(OH)D in 3.2 mm punches from the DBSSs [24,25] using a modified version of a high sensitive liquid chromatography tandem mass spectrometry (LC-MS) method presented by Eyles et al. [24]. The laboratory researchers at SSI that analyzed the samples were blinded for both season of birth and outcome. The coefficient variability for intra-assay and inter-assay for 25(OH)D ranged 7–12% and 7–20%, respectively; and for 25(OH)D2 ranged from 4 to 8% and 9 to 18%, respectively. For all measured concentrations for both intra-assay and inter-assay analysis, there was acceptable precision. The lower limit of quantification (LLOQ) was 4 nmol/L for 25(OH)D and 3 nmol/L for 25(OH)D2, based on empirical observations during the method development. In the present study, the 25(OH)D2 concentrations were omitted, as 89.04% of the 25(OH)D2 were below the LLOQ. Thus only 25(OH)D concentrations, both above (85.16%) and below LLOQ (14.84%), were included in the analysis.

Dried blood spot analysis provides whole blood concentrations, whereas vitamin D is conventionally reported as serum concentrations. In this study, 25(OH)D concentrations are presented in nmol/L, and corrected to report concentrations equivalent to serum concentrations by using the following formula: Serum (25(OH)D) nmol/L = DBSS (25(OH)D) nmol/L × 1/[1−0.61 (the haematrocit fraction)] [22]. We used the haemotrocit fraction for time of birth [22] as the haemotrocit fraction was not measured.

### 2.3. Covariates and Data Sources

Covariates were selected a priori and retrieved from the Danish health care registers. Information on the infant’s sex (female, male) and date of birth was obtained from CRS [18]. Based on date of birth, the variable season of birth (Winter; November–April, Summer; May–October) was created. Information on the infant’s birth weight (<2500, 2500–4000, ≥4000 g) and gestational age (preterm < 37 weeks, term ≥ 37 weeks); and information on maternal age (in years), and parity (primiparous, multiparous) were obtained from the Danish Medical Birth Registry (MBR) [26]. Information on maternal education (elementary school, high school, university) and ethnicity (Western, non-Western) was obtained from Statistics Denmark.

### 2.4. Power Calculation

A power calculation was done before study start of the D-tect study based on the main outcomes (fractures and Type 1 Diabetes) performed under three scenarios related to the fraction of the exposure variance explained including covariates: no adjustment, 30%, or 60% explained. The least detectable hazard ratios (HR) of outcome related to 1 SD difference in 25(OH)D for a sample size of 1000 cases and 1000 controls using α = 0.05 and β = 0.80 were as followed: No covariates 1.09 HR; 30% variance explained 1.11 HR; 60% variance explained 1.15 HR [25]. A power calculation was not done for the outcome in this study since we included all RA cases from the NPR.

### 2.5. Statistical Analysis

Characteristics of the study population stratified by case and subcohort are presented as number (*n*) and percentages (%) for categorical variables and median and interquartile range (IQR) for continuous variables.

**Figure 2:**
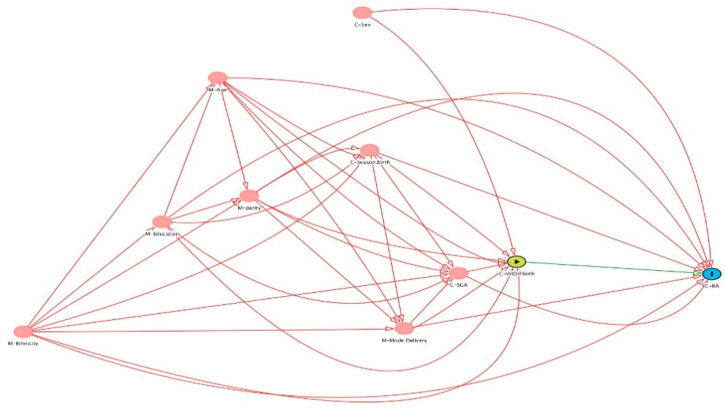
A priori identification of potential confounder through a directed acyclic graph (DAG). C: child, M: Maternal.

A weighted Cox regression analysis, where cases and non-cases were weighted with their inverse sampling probabilities, was used. Using the Cox proportional hazard model with age as the underlying time variable, stratified by year of birth, we assessed the hazard of first RA diagnoses between ages 18 and 33 years and 11 months (33.9 years) by quintiles of 25(OH)D, to capture a potential nonlinear relationship, using the first quintile as reference. The results are presented as hazard ratio (HR) and 95% confidence interval (CI). In the adjusted model, we adjusted for potential confounders identified a priori using a directed acyclic graph (DAG), which were offspring sex, birth weight, preterm birth, maternal age, maternal ethnicity, maternal education, and parity (Figure 2). We tested for overall (no) association using Wald tests.

To address any potential sources of bias, we investigated if individuals included or excluded differed according to baseline variables using chi-square test and t-test or equality of medians test. We further conducted sensitivity analyses by sex and individuals diagnosed after 2003, since private hospitals and outpatient clinics registration in the NPR became mandatory in 2003. We further ran a sensitivity analysis where a potential effect of day of birth was modelled using a cosinor with a yearly period, to take seasonal variation in vitamin D exposure into account.

Statistical analyses were carried out in Stata version 15 (StataCorp LP, College Station, TX, USA). A two-sided *p*-value of <0.05 was considered significant.

### 2.6. Ethical Considerations

Permissions were granted by the Ethical Committee of the Capital Region of (J.no.:H-3-2011-126), the Danish Data Protection Agency (J.no.:2012-41-1156), and permission to use registered data was provided by the Danish Health Data Authority and Statistics Denmark. Permission to access and analyze the DBSSs was granted by the steering committee for scientific use of the Biological Specimen Bank for Neonatal Screening.

## 3. Results

Characteristics of the 805 RA cases with onset in early adulthood and the 2416 individuals from the random subcohort are presented in Table 1. Overall, the median 25(OH)D concentrations was 24.9 nmol/L (IQR 15.4; 36.9) among the cases and 23.87 nmol/L (IQR 13.6; 36.4) among the individuals from the subcohort. In the case group, 75% were women, while in the random subcohort only 49% were women. In addition, for both case and subcohort groups, the majority of the individuals were born at term and had Western origin.

Table S2 presents the characteristics of those included (*n* = 3221) in the analysis and those excluded (*n* = 77) from the analysis due to missing information on covariates. The 25(OH)D concentrations were similar in the two groups, however maternal education differed with more having a ‘elementary school’ or ‘unknown’ education among excluded individuals.

The HR for developing RA between the ages of 18 and 33.9 years was higher in the higher quintiles of vitamin D, however not significant; crude HR: 1.12 (0.85–1.48) and adjusted HR: 1.21 (0.90–1.63) (Table 2). Similar estimates were found in analyses stratified by sex (Table 3). In addition, no significant interaction between vitamin D and sex in relation to RA development was seen (Table S3). In the sensitivity analysis we further (1) included diagnosed after 2003, only and (2) investigated day of birth as a proxy for vitamin D sun exposure. The results were similar to the main analysis and non-significant (Table S3).

## 4. Discussion

Several important factors related to the immune system start developing during the first trimester of pregnancy [27]. Evidence suggests that low vitamin D in early life impairs the development of the immune system [13,14]. Moreover, impaired maternal vitamin D concentrations during pregnancy does not only influence fetal vitamin D concentrations, but it has also been shown to influence the spectrum of immune cells, such as regulatory T cells, in maternal and cord blood [15]. This may have programming effects, in terms of immune tolerance in the fetus, which potentially could affect the risk of developing autoimmune diseases, such as RA, later in life. However, in this large population-based case-cohort study the results did not support the hypothesis that low concentrations of vitamin D at birth were associated with increased risk of RA in early adulthood. The results are similar to those of a large cohort from the UK, which did not find an association between season of birth (as a proxy of vitamin D) and risk of RA [12]. In relation to other autoimmune diseases, we used the same study design and did not find an association between 25(OH)D concentrations around the time of birth with later type 1 diabetes risk, either [28].

Unlike the present study, most research linking vitamin D with RA has focused on vitamin D concentrations around or after disease onset and its relation with disease severity or activity [29]. In recent years, some systematic reviews and/or meta-analysis on the subject have been performed and published [30,31,32]. One review showed that RA patients had lower serum vitamin D than healthy controls and that there was an inverse association between serum vitamin D and RA disease activity among RA patients [31]. Furthermore, in 2017, a systematic review and meta-analysis identified five randomized controlled trials that investigated the effect of at least 3 months of vitamin D supplementation or its analogs on the activity of RA [32]. In this study, the outcomes for RA were recurrence based on Disease Activity Score for RA (DAS-28); reduction in Visual Analog Scale (VAS); and recurrence of disease. There was no significant association between vitamin D and VAS reduction and DAS-28 reduction, also vitamin D produced a non-significant reduction of recurrence (risk difference= −0.10, 95% CI = −0.21;0.00, *p* = 0.05) [32]. On the other hand, a meta-analysis from 2012 reported, based on 10 of the 11 included cohort and supplementation studies, that low vitamin D intake was associated with an increased risk of RA and a greater disease activity [30,33]. Finally, Harrison et al. published a review in 2020 [30] which noted that, overall, there was considerable heterogeneity between studies and their findings, and that most of the evidence was derived from observational rather than RCT studies [30].

Evidence seems to indicate that vitamin D plays a role in the pathophysiology of autoimmune diseases, such as RA [30]. However, the underlying mechanisms and its direction remains unclear [30,34]. It has been demonstrated that vitamin D participates in the regulation of both the innate and adaptive immune system through the vitamin D nuclear receptor (VDR) [35,36]. A murine model of chronic arthritis, in which mice lacking VDR were interbred with human tumor necrosis factor (TNF) transgenic mice, showed that the absence of VDR exacerbated inflammation and the severity of arthritis [37]. In addition, a recent systematic review concluded that there was an association between VDR gene polymorphisms and risk of RA, and that different polymorphisms may increase or decrease RA risk in different populations [38].

Overall, evidence also seems to indicate that vitamin D plays an important role in regulating T-cells to limit immune mediated diseases [39].

This study has strengths and limitations that need to be considered when interpreting the results. The inclusion of all RA cases and the use of neonatal biomaterial are its two main strengths. This study included all cases of RA from the entire population born in Denmark from 1981 to 1996 and who had DBSSs available and the random subcohort was sampled conditional on year of birth, from the same period. However, a power calculation was not performed before study start since all RA cases were included, thus the results might have been underpowered. It has been shown that measured 25(OH)D from DBSSs provides a valid and reliable alternative to the conventional methods that use sera or plasma [40]. Another strength of this study is that it was possible to adjust for several potential confounders available in the Danish health care registers, which have high quality and cover the entire population [19,41]. However, we cannot exclude residual confounding from variables that we lacked information on, such as information on nutritional or lifestyle factors during pregnancy. Nevertheless, we adjusted for education, which may be highly correlated with potential residual confounders [42].

A potential limitation is that data on RA diagnose was extracted from the NPR and based on the ICD-10 codes recorded by physicians at in- and outpatient somatic and psychiatric care units irrespective of specialty. Therefore, a selection towards more severe cases of RA cannot be excluded, as the results will not account for patients that are exclusively diagnosed and treated in primary care units, which would have attenuated our results and contributed to the finding of insignificant results. In addition, we cannot exclude misclassification of cases, which in this study were identified based on the first-time record of an ICD code for RA during 1999–2015. Pedersen et al. aimed to provide measures of validity and completeness of RA diagnoses recorded in the NPR [43]. The authors found that only 59% of RA cases (*n* = 217) registered in NPR between 1977 and 2001 could be confirmed as RA cases by a review of the medical records; however, the proportion increased when registered inpatients were included (up to 80%), as in in the present study, and when having more than one registration of RA diagnosis (up to 91%) [43]. Moreover, in recent years, medicine in general has progressed considerably, including the field of rheumatology, thus we expect that the validity and completeness of RA diagnoses has improved further since 2001.

It is well-known that the prevalence of RA increases with age [44]. In this study, the RA cases were limited to cases with RA onset in early adulthood (18–33.9 years) only, which means that this study examined the influence of neonatal vitamin D on early onset RA. This lack of association could thus also be attributed to the rather late peak onset of RA pointing to adult immune modulation, environmental, and nutritional factors in later life as being more important for the triggering of RA.

We had only one measurement of 25(OH)D, which was collected up to a week after birth, and thus likely reflecting 25(OH)D concentrations during the last months of pregnancy. Vitamin D measurements during the first or second trimesters could have yielded a different result since these trimesters are important periods for the development and maturation of the immune system.

Lastly, 25(OH)D concentrations in this study were considerably lower than concentrations quantified in another study with a similar population, which also measured 25(OH)D from DBSSs using the same laboratory as we did (for all subjects: median 32.3, IQR 20.5–46.6) [45]. In addition, 14.8% of the concentrations of 25(OH)D were below the LLOQ. Therefore, we reported quintiles of 25(OH)D concentrations with arbitrary cut-offs, and not the recommended standard cut-offs [46], which makes it difficult to compare concentrations in this study with other studies. In this study, the storage time of DBSSs was up to almost 34 years; however, it is unlikely that the low concentrations observed in our study are due to sample degradation, as it has been shown that storage times of 25(OH)D for more than 20 years are stable, regardless of storage temperature and light exposure [24]. In addition, such bias would affect similar studies [45]. Seasonal variations in 25(OH)D concentration from DBSS were well captured; hence it seems unlikely that the low 25(OH)D quantified in this study have biased the ranking of individuals into quintiles of concentrations.

In conclusion, no association between neonatal 25(OH)D concentrations and the risk of developing RA in early adulthood (18–33 years of age) was observed in this study. Future studies should focus on the two first trimesters of pregnancy and infancy, which are important periods for the development and maturation of the immune system.

## Figures and Tables

**Table 1:** Characteristics of the study population.

	Cases	Random Subcohort	*p*-Value
N	805	2416	
25(OH)D nmol/L median [IQR]	24.9 (15.4; 36.9)	23.9 (13.6; 36.4)	0.15
Case *n* (%)			
No	0 (0.0)	2414 (99.9)	
Yes	805 (100.0)	2 (0.1)	
Offspring sex *n* (%)			<0.001
Male	201 (25.0)	1234 (51.1)	
Female	604 (75.0)	1182 (48.9)	
Maternal ethnicity *n* (%)			0.011
Western	784 (97.4)	2303 (95.3)	
Non-Western	21 (2.6)	113 (4.7)	
Maternal age in years median [IQR]	27 (24; 30)	27 (24; 31)	0.04
Maternal education *n* (%)			0.015
Elementary school	313 (38.9)	843 (34.9)	
Highschool	320 (39.8)	1040 (43.1)	
University	138 (17.1)	468 (19.4)	
Unknown	34 (4.2)	65 (2.7)	
Parity *n* (%)			0.60
Primiparous	376 (46.7)	1103 (45.7)	
Multiparous	429 (53.3)	1313 (54.4)	
Season of birth *n* (%)			0.98
Winter	405 (50.3)	1214 (50.3)	
Summer	400 (49.7)	1202 (49.8)	
Gestational age *n* (%)			0.73
≥37 weeks	756 (93.9)	2277 (94.3)	
<37 weeks	49 (6.1)	139 (5.8)	
Birth weight in grams median [IQR]	3450 (3150; 3800)	3470 (3128; 3810)	0.39
Maternal RA *n* (%)			<0.001
No	694 (86.2)	2220 (91.9)	
Yes	111 (13.8)	196 (8.1)	
Paternal RA *n* (%)			0.10
No	714 (88.7)	2191 (90.7)	
Yes	91 (11.3)	225 (9.3)	

**Table 2:** Unadjusted and adjusted hazard ration (95% CI) of RA cases in early adulthood among Danish individuals aged 18–33 years, according to quintiles of neonatal 25(OH)D concentrations.

Quintiles Limit, (nmol/L)	Crude (*n* = 3221)	Adjusted ^a^ (*n* = 3221)
Q1 [0–12.1]	1 (ref)	1 (ref)
Q2 [12.1–19.6]	0.89 (0.67–1.18)	0.89 (0.66–1.19)
Q3 [19.6–28.0]	1.06 (0.80–1.40)	1.13 (0.84–1.52)
Q4 [28.0–40.1]	1.15 (0.88–1.51)	1.17 (0.88–1.57)
Q5 [40.1–114.9]	1.12 (0.85–1.48)	1.19 (0.88–1.60)

^a^: offspring sex, birth weight, preterm birth, maternal age, maternal ethnicity, maternal education, parity.

**Table 3:** Unadjusted and adjusted hazard ration (95% CI) of RA cases in early adulthood among Danish male and female individuals aged 18–33 years, according to quintiles of neonatal 25(OH)D concentrations.

Quintiles	Male		Female	
	Crude (*n* = 1435)	Adjusted ^b^ (*n* = 1435)	Crude (*n* = 1786)	Adjusted ^b^ (*n* = 1786)
Q1	1 (ref)	1 (ref)	1 (ref)	1 (ref)
Q2	0.91 (0.54–1.55)	0.96 (0.54–1.70)	0.85 (0.60–1.21)	0.84 (0.59–1.20)
Q3	0.97 (0.58–1.62)	0.97 (0.56–1.67)	1.10 (0.60–1.56)	1.12 (0.78–1.60)
Q4	1.39 (0.86–2.27)	1.46 (0.88–2.45)	1.10 (0.83–1.66)	1.20 (0.84–1.71)
Q5	1.25 (0.76–2.05)	1.31 (0.75–2.28)	1.05 (0.74–1.48)	1.06 (0.74–1.52)

^b^: birth weight, preterm birth, maternal age, maternal ethnicity, maternal education, parity.

## Data Availability

Data is available on a secure platform on Statistics Denmark’s homepage. To get access to data, an application has to be sent to The Parker Institute and permission has been granted by the Danish Data Protection Agency, Statistics Denmark, and the Ethical Committee of the Capital Region.

## Supplementary Materials

The following supporting information can be downloaded at: https://www.mdpi.com/article/10.3390/nu14030447/s1, Table S1: STROBE Statement—Checklist of items that should be included in reports of case-control studies; Table S2: Demographic information for included and excluded individuals; Table S3: Sensitivity analysis investigating 1. Interaction effects among vitamin D and sex, 2. Cosinor analysis of day at birth as a proxy for seasonal variation I vitamin D exposure, and 3. Analysis of individuals with RA diagnosis after 2003.
