# Supplementary material for: Vitamin D Concentrations at Birth and the Risk of Rheumatoid Arthritis in Early Adulthood: A Danish Population-Based Case-Cohort Study
Source: Nutrients. 2022 Jan 20;14(3):447. doi: 10.3390/nu14030447 (PMC8839203; doi:10.3390/nu14030447)
Supplement: Supplementary file 1 [file nutrients-14-00447-s001.zip › nutrients-1567124-supplementary.pdf]

Table S1. STROBE Statement—Checklist of items that should be included in reports of ***case-control studies***

	Item No	Recommendation	Page No
**Title and abstract**	1	(*a*) Indicate the study's design with a commonly used term in the title or the abstract	1
		(*b*) Provide in the abstract an informative and balanced summary of what was done and what was found	3
**Introduction**			
Background/rationale	2	Explain the scientific background and rationale for the investigation being reported	4
Objectives	3	State specific objectives, including any prespecified hypotheses	4
**Methods**			
Study design	4	Present key elements of study design early in the paper	5
Setting	5	Describe the setting, locations, and relevant dates, including periods of recruitment, exposure, follow-up, and data collection	5
Participants	6	(*a*) Give the eligibility criteria, and the sources and methods of case ascertainment and control selection. Give the rationale for the choice of cases and controls	5
		(*b*) For matched studies, give matching criteria and the number of controls per case	NA
Variables	7	Clearly define all outcomes, exposures, predictors, potential confounders, and effect modifiers. Give diagnostic criteria, if applicable	5-7
Data sources/ measurement	8*	For each variable of interest, give sources of data and details of methods of assessment (measurement). Describe comparability of assessment methods if there is more than one group	5-7
Bias	9	Describe any efforts to address potential sources of bias	7
Study size	10	Explain how the study size was arrived at	Flow chart p. 8
Quantitative variables	11	Explain how quantitative variables were handled in the analyses. If applicable, describe which groupings were chosen and why	6-7
Statistical methods	12	(*a*) Describe all statistical methods, including those used to control for confounding	6-7
		(*b*) Describe any methods used to examine subgroups and interactions	7
		(*c*) Explain how missing data were addressed	5
		(*d*) If applicable, explain how matching of cases and controls was addressed	NA
		(*e*) Describe any sensitivity analyses	7
**Results**			
Participants	13*	(a) Report numbers of individuals at each stage of study—eg numbers potentially eligible, examined for eligibility, confirmed eligible, included in the study, completing follow-up, and analysed	Flow chart p. 8
		(b) Give reasons for non-participation at each stage	Flow chart p. 8
		(c) Consider use of a flow diagram	Flow chart p. 8

Descriptive data	14*	(a) Give characteristics of study participants (eg demographic, clinical, social) and information on exposures and potential confounders	9 and table 1
		(b) Indicate number of participants with missing data for each variable of interest	Sup. Table 1
Outcome data	15*	Report numbers in each exposure category, or summary measures of exposure	9
Main results	16	(*a*) Give unadjusted estimates and, if applicable, confounder-adjusted estimates and their precision (eg, 95% confidence interval). Make clear which confounders were adjusted for and why they were included	9 and table 2
		(*b*) Report category boundaries when continuous variables were categorized	NA
		(*c*) If relevant, consider translating estimates of relative risk into absolute risk for a meaningful time period	NA
Other analyses	17	Report other analyses done—eg analyses of subgroups and interactions, and sensitivity analyses	Table 3 and p. 9
**Discussion**			
Key results	18	Summarise key results with reference to study objectives	12
Limitations	19	Discuss limitations of the study, taking into account sources of potential bias or imprecision. Discuss both direction and magnitude of any potential bias	13
Interpretation	20	Give a cautious overall interpretation of results considering objectives, limitations, multiplicity of analyses, results from similar studies, and other relevant evidence	14
Generalisability	21	Discuss the generalisability (external validity) of the study results	14
**Other information**			
Funding	22	Give the source of funding and the role of the funders for the present study and, if applicable, for the original study on which the present article is based	2

Table S2. Demographic information for included and excluded individuals.

	**Included**	**Excluded**	**p-value**
n	3221	77	
25(OH)D_3 nmol/L *median [IQR]*	24.87 [15.38, 36.92]	21.74 [12.62, 33.08]	0.43
Offspring sex *n (%)*			0.70
Male	1435 (44.6)	36 (46.8)	
Female	1786 (55.5)	41 (53.3)	
Maternal ethnicity *n (%)*			<0.001
Western	3087 (95.8)	48 (62.3)	
Non-Western	134 (4.2)	5 (6.5)	
Missing	0	25 (31.2)	
Maternal age in years *median [IQR]*	27.0 [24.0, 31.5]	26.0 [23.5, 31.5]	0.43
Maternal education *n (%)*			<0.001
Elementary school	1156 (35.5)	14 (18.2)	
Highschool	1370 (41.5)	10 (13.0)	
University	614 (18.6)	8 (10.4)	
Unknown	99 (3.1)	45 (58.4)	
Parity *n (%)*			0.061
Primiparous	1479 (45.9)	20 (62.5)	
Multiparous	1742 (54.1)	12 (37.5)	
Season of birth *n (%)*			0.33
Winther	1619 (50.3)	43 (55.8)	
Summer	1602 (49.7)	34 (44.2)	
Gestational age *n (%)*			0.73
≥37 weeks	3033 (94.2)	2 (100.0)	
<37 weeks	188 (5.8)	0 (0)	
Birth weight in grams *median [IQR]*	3460 [3140, 3800]	3250 [3000, 3550]	0.09
Maternal RA *n (%)*			0.37
No	2914 (90.5)	72 (93.5)	
Yes	307 (9.5)	5 (6.5)	
Paternal RA *n (%)*			0.08
No	2905 (90.2)	74 (96.1)	
Yes	316 (9.8)	3 (3.9)	

Table S3. Sensitivity analysis investigating 1. interaction effects among vitamin D and sex, 2. cosinor analysis of day at birth as a proxy for seasonal variation I vitamin D exposure, and 3. analysis of individuals with RA diagnosis after 2003.

	Interaction analysis between vitamin D and sex			Cosinor analysis of day of birth			Starting follow-up after 2003					
**Quintiles limit**	**HR**	**95% CI**		**HR**	**95% CI**		**HR crude**	**95% CI**		**HR adjusted[1]**	**95% CI**	
Q1	1	-	-	1	-	-	1	-	-	1	-	-
Q2	0.96	0.51	1.51	0.88	0.65	1.18	0.88	0.66	1.18	0.88	0.65	1.19
Q3	1.17	0.63	2.15	1.11	0.83	1.50	1.07	0.81	1.42	1.15	0.85	1.55
Q4	0.86	0.48	1.55	1.15	0.85	1.56	1.15	0.87	1.51	1.18	0.88	1.58
Q5	0.09	0.47	1.55	1.16	0.84	1.60	1.11	0.84	1.47	1.18	0.87	1.60

[1] adjusted for offspring sex, birth weight, preterm birth, maternal age, maternal ethnicity, maternal education, parity
